# Searching for the Least Invasive Management of Pelvi-Ureteric Junction Obstruction in Children: A Critical Literature Review of Comparative Outcomes

**DOI:** 10.3389/fped.2020.00252

**Published:** 2020-06-02

**Authors:** Marco Castagnetti, Massimo Iafrate, Ciro Esposito, Ramnath Subramaniam

**Affiliations:** ^1^Section of Paediatric Urology, Department of Surgical, Oncological, and Gastrointestinal Sciences, University Hospital of Padova, Padua, Italy; ^2^Department of Paediatrics, Federico II University of Naples, Naples, Italy; ^3^Department of Paediatric Urology, Leeds Teaching Hospitals NHS Trust, University of Leeds, Leeds, United Kingdom; ^4^Department of Paediatric Urology, University of Ghent, Ghent, Belgium

**Keywords:** pyeloplasty, pelvi-ureteric junction, obstructive uropathy, hydronephrosis, minimally-invasive surgery, robotic surgery

## Abstract

**Introduction:** To review the published evidence on the minimally invasive pyeloplasty techniques available currently with particular emphasis on the comparative data about the various minimally invasive alternatives to treat pelvi-ureteric junction obstruction and gauge if one should be favored under certain circumstances.

**Materials and Methods:** Non-systematic review of literature on open and minimally invasive pyeloplasty including various kinds of laparoscopic procedures, the robotic-assisted laparoscopic pyeloplasty, and endourological procedures.

**Results:** Any particular minimally invasive pyeloplasty procedure seems feasible in experienced hands, irrespective of age including infants. Comparative data suggest that the robotic-assisted procedure has gained wider acceptance mainly because it is ergonomically more suited to surgeon well-being and facilitates advanced skills with dexterity thanks to 7 degrees of freedom. However, costs remain the major drawback of robotic surgery. In young children and infants, instead, open surgery can be performed via a relatively small incision and quicker time frame.

**Conclusions:** The best approach for pyeloplasty is still a matter of debate. The robotic approach has gained increasing acceptance over the last years with major advantages of the surgeon well-being and ergonomics and the ease of suturing. Evidence, however, may favor the use of open surgery in infancy.

## Introduction

Open pyeloplasty has been for ages considered the gold standard treatment of pyelo-ureteric junction (PUJ) obstruction, and the standards of open pyeloplasty were set back in 1998 by Gerard Monfort (1). Using optical magnification and fine suture materials, it has been shown that the procedure can be performed as a day case surgery, without any indwelling stent, with >95% success rate. Long-term durability of open pyeloplasty has also been well-documented (2).

Despite the good outcomes of open pyeloplasty, the search for less invasive treatment modalities alternative to open pyeloplasty has continued. The potential advantages of a minimally invasive approach for the dissection have never been questioned; the main hurdle lies with the accomplishment of the pyelo-ureteral anastomosis that can require advanced suturing skills and can be time-consuming even in experienced hands, a fact particularly true with laparoscopic techniques (3). Consistently, in a systematic review and meta-analysis of open vs. minimally invasive pyeloplasty (MIP) performed in 2014, Autorino et al. observed that although MIP procedures can achieve complication and success rates comparable to open surgery, the operating time still largely favors open pyeloplasty (4). More importantly, multiple reports coming for different institutions prove that open pyeloplasty is safe and duplicable in the widespread use, and duplicability of the MIP procedure is more controversial as the skills necessary to perform the procedure can be hard to achieve and maintain (5). The most complex scenario is clearly that of a pyeloplasty performed in an infant (6), which is not an uncommon scenario with prenatal diagnosis, the most common presentation of PUJ obstruction, and most of these patients who require surgery do so in infancy (7).

The aim of the present review was to summarize the available evidence on the MIP techniques currently available with particular emphasis on the comparative data about the various MIP alternatives to gauge if one should be favored under certain circumstances.

## MIP Techniques

MIP is an umbrella term that encompasses several techniques including laparoscopic surgery and robotic-assisted laparoscopic pyeloplasty (RALP) and can be performed using a trans-peritoneal or a retro-peritoneal route. The standard robotic instruments are 8 mm with cable-driven hinges, and although 5-mm instruments with metal hinges are available, the range of movements is difficult to realize especially in limited space. Traditional laparoscopic approach can be achieved with a 5-mm camera and 3-mm instruments, also referred to as “Mini-laparoscopy.” Other recognized approaches include single-site surgery or one-trocar-assisted pyeloplasty (OTAP).

Single-site surgery also known as LESS (laparo-endoscopic single site) surgery is performed introducing all the instruments necessary to perform the procedure via a single umbilical incision, with or without a specific device (8). In the OTAP, instead, the dissection is performed laparoscopically using a retroperitoneal approach, whereas the PUJ is delivered outside the abdomen to perform the pyeloplasty externally like in open surgery (9). This procedure potentially combines the putative advantages of both a minimally invasive dissection and an easier open pyeloplasty keeping the incision small at the same time. The major limitation of the OTAP is patient size, as delivery of the PUJ can possibly be difficult in older patients.

In terms of the procedure, dismembered Anderson–Hynes pyeloplasty is the standard technique of choice. In MIP, this procedure requires advanced skills of suturing, which some surgeons find tedious and not comfortable ergonomically (10, 11). In order to circumvent the problems related to the suturing skills necessary to perform the procedure minimally invasively, in recent years, interest has increased with alternatives, such as the vascular hitch for PUJ obstructions due to extrinsic compression by a crossing vessel (12), or non-dismembered pyeloplasty for intrinsic PUJ obstructions (13). RALP is definitely the superior approach facilitating advanced suturing skills in MIP although cost is the main prohibitive factor preventing widespread acceptance as alluded to later on in this article.

Endourological techniques can also be considered minimally invasive modalities to treat PUJ obstruction. These include a range of procedures, such as the balloon dilatation of the PUJ and the endopyelotomy (14, 15) with availability of cutting balloons combining dilatation and endopyelotomy (16). Any endourological procedures can be performed using a retrograde or antegrade approach.

## Single-Institution Results

For any of the mentioned minimally invasive treatment modalities, single-surgeon or single-institution series exist documenting feasibility and effectiveness (Table 1). The procedure can be carried out successfully at any age including infancy, although it is clearly more demanding in small patients given the small available operating space (20). Only endourological techniques are probably an exception; even in the most experienced hands, reported failure rate is 2- to 3-fold higher than the other techniques (Table 1). Consistently, a systematic review published in 2015 shows that this treatment modality has not gained wide acceptance (only 128 cases reported) and the complication rate (14.8%) is much higher and the median success rate (71%) is much lower than those reported for MIP (15). Nevertheless, for all the MIP techniques, duplicability and cost-effectiveness remain to be proven and we still need comparative data to assess which technique is more effective and under which circumstances.

**Table 1:** Single institution series on minimally invasive treatment of pelvi-ureteric junction obstruction.

**Series**	**Technique**	**N of Pts**	**Conversion rate**	**Failure rate**
Chandarasekaram (17)	Laparoscopy	111	0	1%
Blanc et al. (18)	Retroperitoneoscopy	104	3%	2%
Lima et al. (9)	OTAP	155	8%	1%
Minnillo et al. (19)	RALP	155	0	3%
Parente et al. (14)	Baloon dilatation	50	0	10%

## Comparative Data on MIP Procedures

An analysis of the published literature regarding RALP shows that despite the constantly increasing number of publications over years, the level of evidence for available studies remains limited to case reports, case series, and retrospective comparative studies (21). This issue, however, is unfortunately true for any MIP procedure (21).

In terms of comparative data, we have studies comparing laparoscopy pyeloplasty vs. endourological management, laparoscopic vs. retroperitoneoscopic pyeloplasty, and laparoscopic vs. robot-assisted pyeloplasty.

In the single series comparing retrograde balloon dilatation and laparoscopic pyeloplasty, balloon dilatation had a significantly shorter operating time and hospital stay, and significantly lower analgesic requirement and costs (22). The study confirms, however, that the real issue with the endourological techniques is the success rate, particularly in the long term. Balloon dilatation seems not to be a durable procedure. Both procedures indeed had comparable success rate at 3 months, 94.7% for balloon dilatation vs. 97.1% of laparoscopic pyeloplasty, but the success rate of balloon dilatation progressively dropped to 71% at 2 years follow-up, becoming significantly lower than laparoscopic pyeloplasty, the success rate for which instead remained pretty steady over time (22).

The comparison of laparoscopic vs. retroperitoneoscopic pyeloplasty has been the objective of one of the few randomized clinical trials available in pediatric urology. Badawy et al. compared 19 patients randomized to each MIP approach (23). Success rate was comparable, whereas the retroperitoneal approach had shorter operative time by an average 40 min with earlier resumption of oral feeding and, as a consequence, shorter hospital stay. These data are in contrast with those of what is probably the largest single surgeon series of pyeloplasty available in the literature by Liu et al. (8). This series includes 1,750 pyeloplasties, 451 retroperitoneoscopic, 311 laparoscopic, 322 LESS, and 805 trans-umbilical multiport. The two approaches had comparable complication and success rate in both these reports, with the retroperitoneoscopic approach having quicker resumption of oral feeding and shorter hospital stay. However, in the latter series (8), the complication rate was higher and operative time was significantly longer for retroperitoneoscopy than any other MIP procedure in contrast to the report by Badawy et al. (23). These results are consistent with a meta-analysis of one randomized clinical trial and eight clinical trials (776 participants) in adults (24). In summary, these data suggest that the trans-peritoneal approach may be easier to perform while the creation of a retroperitoneal working chamber might increase the complexity of the procedure, making it longer and increasing the risk of conversion. Potential disadvantages of trans-peritoneal route include a longer post-operative ileus, the risk of intraperitoneal urine leakage post-operatively and adhesions formation in the long term.

The comparison between laparoscopic and RALP is the one that has attracted more attention in the recent past. Since 2009, four systematic reviews and meta-analyses have been published on this topic (4, 25–27). The most recent one includes 14 studies: 1 prospective trial, 1 case–control, and 12 retrospective series (27). Once again, the general level of evidence is low, but the quality of studies was quite good with a low risk of bias in 10 out of 14. The meta-analysis showed that the operating time was equivalent, whereas all the other outcome parameters including hospital stay, complication rate, success rate, and re-intervention rate favored or tended to favor the robotic-assisted procedure.

**Figure 1:**
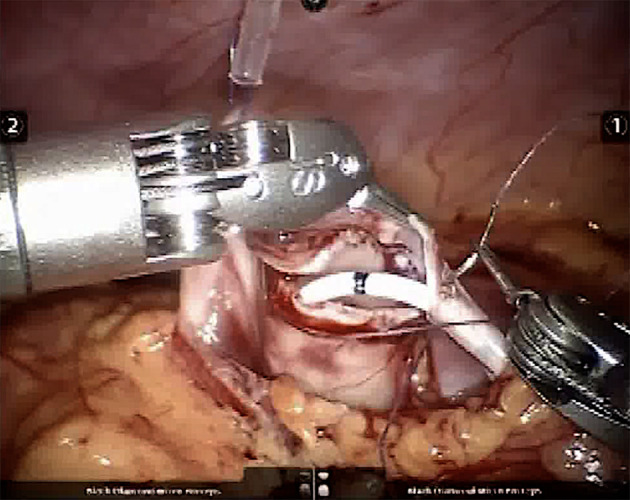
Intraoperative picture showing the potential for articulation of the robotic instruments, which greatly simplifies suturing.

This is consistent with the putative advantages of the robotic approach, including comfortable position for the surgeon, 3D view, and steady instruments with 7 degrees of freedom that make suturing much easier (Figure 1). It sounds reasonable that operating in a more comfortable way allows better results. It is well-documented that long-lasting laparoscopic procedures might cause chronic musculo-skeletal discomfort to the surgeon (11).

Consistently with this observation, Varda et al., analyzing the trend in utilization of open, laparoscopic, and robotic pyeloplasty in the United States from 2003 to 2015, reported since 2004, when the Da Vinci system became available, that the number of MIP procedures has progressively increased, mainly due to an increase in the number of robotic-assisted procedures, whereas the number of laparoscopic procedure has progressively decreased (28). Although not considered in the meta-analyses, another potential advantage of the robotic approach over conventional laparoscopy is that its learning curve is less steep (29), and since the use of robotic surgery further limits the volume of cases undergoing laparoscopic surgery, it is likely that the increased use of robotic surgery will permanently limit the use of conventional laparoscopic pyeloplasty in children, in the centers where the robot is available.

## Robotic-Assisted Laparoscopic Pyeloplasty

The two major drawbacks of robotic-assisted pyeloplasty include costs and the size of the instruments.

Varda et al. estimated costs of open, laparoscopic, and robotic pyeloplasty and noted that the latter has a significantly higher cost mainly due to the cost of the consumables (28). This model, however, does not take into account the cost of the robot itself. Using a mathematical model, Behan et al. estimated that, in a center performing 100 RALP per year, the cost of the robot would not be neutralized even after 10 years (30).

Costs can be reduced using appropriate strategies. The first step is to reduce the console time and operating room turnover. Seideman et al. estimated that with a 2-days in-stay, RP could be cost-effective (when compared with LP) if it was carried out in under 120 min (31). Console time normally decreases with increasing experience and progression in the learning curve. It should be noted, however, that trainee involvement with the robot may make it difficult to lower console time as fellows and residents turnover regularly (32). Having a team specialized and dedicated just to robotic cases, instead, can reduce turnover time, particularly docking and undocking time (32, 33).

Increased and regular utilization of the robot by multiple services, i.e., increasing the volume of robotic procedures, is another important cost-saving strategy (32, 33).

Finally, increasing competition within the industry could translate into the end of the current monopoly, which could then translate to steadily reduce the cost of the robotic equipment, making robotics a more cost-effective and affordable technique (33).

The other issue is the size of the robotic instruments. The most modern standard instruments are 8 mm in size and also the arms of the robot are cumbersome. Smaller, 5-mm instruments do exist (34), but they have a pulley system that limits articulation and precludes certain movements. For this reason, many surgeons recommend the routine use of 8-mm instruments for all pediatric cases irrespective of age (35).

Consistently, splitting the results reported by Varda et al. by the age of the patients undergoing pyeloplasty, it is apparent that the use of the RALP mainly increased in the adolescent age group (13–18 years), whereas its use was very limited in infants (28).

## Pyeloplasty in Infants

**Figure 2:**
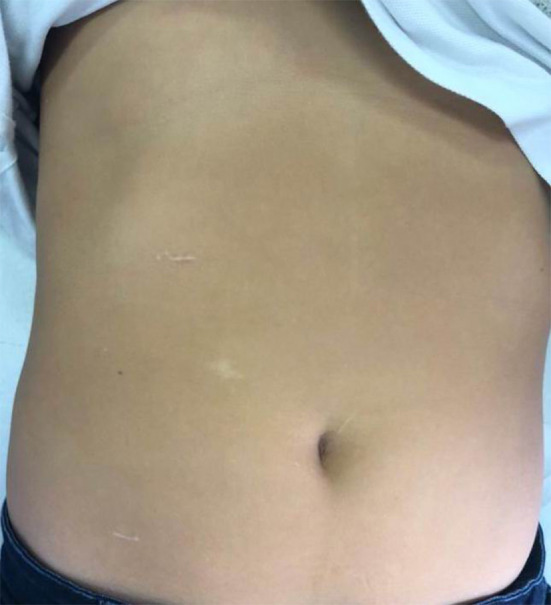
Scar appearance after laparoscopic pyeloplasty.

In the era of antenatal diagnosis of hydronephrosis, the infantile group represents an important age group for surgery. In this group of patients, RALP is feasible, but its role seems limited and has not gained wide acceptance. One issue is the size of the instruments mentioned above. In this, the use of 3-mm instruments, the so called mini-laparoscopy, can be advantageous (36) (Figure 2). Nevertheless, the accomplishment of a pyeloplasty in the limited space of an infant abdomen can be extremely demanding. Moreover, in younger patients, MIP does not seem to offer the same advantages in terms of shorter hospital stay and lower narcotic requirements observed instead in pre-adolescent and adolescent patients (37).

The second and perhaps the most relevant issue in this age group is the concern about the potential neurotoxicity of the drugs for the general anesthesia in early childhood (38, 39). For this reason, many authors and scientific societies recommend in this age group, if surgery cannot be postponed, at least the quickest procedure should be preferred. Published evidence overall thus far, as regards operative time, favors open pyeloplasty over MIP procedures (4).

**Figure 3:**
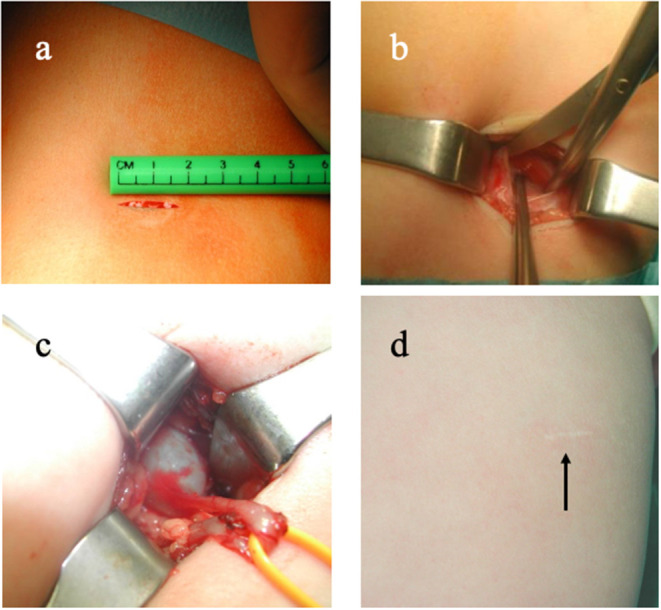
Example of minimally invasive open pyeloplasty. **(a)** 2-cm incision; **(b)** muscle-sparing approach; **(c)** delivery of the pelvi-ureteric junction via the incision; **(d)** barely visible scar 6 months after the procedure.

In this respect, it is relevant that the procedure can be performed via such a small approach in infants to be called “minimally invasive open pyeloplasty” (40–42). Chako et al. reported that in patients <5 years, the procedure can be performed via an incision <3 cm in about 100 min on average combining a quick procedure with good cosmetic outcome (42) (Figure 3). However, some potential limitations of this approach should be considered. A small incision limits exposure of the anatomical structures, which can be an issue in case of unexpected anatomical variants. For this reason, advocates of this approach have underscored the importance of determining the exact incision site by intraoperative renal ultrasonography (40), and/or performing a retrograde pyelogram at the beginning of the surgery to define exactly the PUJ anatomy (41). Otherwise, a minimally invasive approach might prove somewhat more flexible while dealing with unexpected variants. Nevertheless, performing a pyeloplasty in an abnormal kidney and in an infant abdomen remains a formidable endeavor.

## Cosmetic Results of Open VS. MIP

Cosmetic results are a relevant aspect in the decision-making. Gatte et al. performing a randomized, prospective, controlled trial comparing laparoscopic vs. open pyeloplasty concluded that both approaches are comparable and equally effective methods for repair of PUJ obstruction. Although operative time seems statistically shorter in the open group and length of stay seems shorter in the laparoscopic group, the choice should be based on family preference for incision aesthetics and surgeon comfort with either approach, rather than more classically objective outcome measures (43). In this respect, Wang et al. confirmed that larger initial incisions tend to grow more; therefore, at the same follow-up interval, laparoscopic incisions are smaller than those of open procedures (44). Barbossa et al. studied family preferences based on the assessment of pictures and diagrams of the scars of open pyeloplasty and RALP (45). They reported that families prefer the RALP scars both based on pictures and diagrams. Nevertheless, this held true only provided that there was no apparent medical benefit associated with one of the two procedures. Moreover, the approach did not seem to be a statistically significant factor in patients being pleased or not with the scar appearance in the study by Wang et al. (44).

## Conclusions

Any MIP procedure seems feasible in experienced hands, even in infants. The best approach for pyeloplasty is still a matter of debate. The robotic approach seems to have gained increasing acceptance over the last years with major advantages being ergonomics and the ease of suturing. Costs and the size of the instruments remain major drawbacks for the application of the robotic approach in children. Evidence may favor the use of open surgery in infancy.

## Author Contributions

MC and RS drafted the manuscript, reviewed the literature, and also supplied one figure each. MI and CE suggested the articles for review and gave advice during the process of writing the manuscript. CE also supplied one figure.

## Conflict of Interest

The authors declare that the research was conducted in the absence of any commercial or financial relationships that could be construed as a potential conflict of interest.
